# E3 ligases: a ubiquitous link between DNA repair, DNA replication and human disease

**DOI:** 10.1042/BCJ20240124

**Published:** 2024-07-10

**Authors:** Anoop S. Chauhan, Satpal S. Jhujh, Grant S. Stewart

**Affiliations:** Institute of Cancer and Genomic Sciences, College of Medical and Dental Sciences, University of Birmingham, Birmingham, U.K.

**Keywords:** DNA damage response, DNA repair, DNA replication, E3 ligase, human disease, ubiquitin

## Abstract

Maintenance of genome stability is of paramount importance for the survival of an organism. However, genomic integrity is constantly being challenged by various endogenous and exogenous processes that damage DNA. Therefore, cells are heavily reliant on DNA repair pathways that have evolved to deal with every type of genotoxic insult that threatens to compromise genome stability. Notably, inherited mutations in genes encoding proteins involved in these protective pathways trigger the onset of disease that is driven by chromosome instability e.g. neurodevelopmental abnormalities, neurodegeneration, premature ageing, immunodeficiency and cancer development. The ability of cells to regulate the recruitment of specific DNA repair proteins to sites of DNA damage is extremely complex but is primarily mediated by protein post-translational modifications (PTMs). Ubiquitylation is one such PTM, which controls genome stability by regulating protein localisation, protein turnover, protein-protein interactions and intra-cellular signalling. Over the past two decades, numerous ubiquitin (Ub) E3 ligases have been identified to play a crucial role not only in the initiation of DNA replication and DNA damage repair but also in the efficient termination of these processes. In this review, we discuss our current understanding of how different Ub E3 ligases (RNF168, TRAIP, HUWE1, TRIP12, FANCL, BRCA1, RFWD3) function to regulate DNA repair and replication and the pathological consequences arising from inheriting deleterious mutations that compromise the Ub-dependent DNA damage response.

## Introduction

Precise genome duplication and maintenance of its stability are central to all life. Therefore, cells have evolved a dedicated set of proteins and pathways to preserve genomic integrity. In mammalian cells, genome duplication is carried out in three phases; replication initiation, elongation and termination. During initiation, a pre-replicative complex (pre-RC) is formed at replication origins, which involves assembly of the hexameric MCM helicase onto DNA bound by the origin recognition complex [1]. In early S-phase, these licenced origins are fired by the combined actions of both Cyclin-dependent kinases (CDK) and the Dbf4/Drf1-dependent kinase, which facilitate the loading of critical components of the replication machinery e.g. CDC45, TICRR/MTBP, DONSON, TOPBP1, GINS and MCM10 onto the MCM helicase [2]. During the elongation step of replication, synthesis of the daughter strand is carried out by the co-ordinated action of three DNA polymerases namely α, δ, and ε. Pol α in combination with primase catalyses synthesis of the RNA primer and the first 20 nucleotides of the Okazaki fragment. In contrast, Pol δ, and ε in association with various processivity factors e.g. the replication factor C complex and proliferating cell nuclear antigen (PCNA) catalyse synthesis of the lagging and leading strands respectively. Finally, termination occurs when two replication forks converge, which is initiated by the poly-ubiquitylation of components of the MCM helicase and subsequent disassembly of replication machinery by the p97/VCP segregase [3,4].

As replication does not occur in isolation, a moving replication fork encounters various roadblocks that can impede its progression, which can result in fork stalling or premature termination leaving regions of the genome under-replicated. This phenomenon is termed ‘replication stress’ and encompasses any physiological or pathological impediment to the process of DNA synthesis [5]. If not resolved properly, replication stress can cause DNA damage and/or chromosomal segregation defects, which is a persistent threat to genome stability [5]. Additionally, genomic DNA is constantly under attack from various endogenous and exogenous sources of damage e.g. reactive oxygen species and UV-light. These can directly or indirectly damage individual nucleotides, generate single-stranded (SSB) or double-stranded (DSB) DNA breaks, cause inter- or intra-stand DNA cross-links, or form DNA-protein crosslinks (DPCs) [6–8]. If these genomic lesions are not repaired promptly they can obstruct DNA replication, inhibit gene transcription, stimulate the generation of chromosomal translocations and/or prevent faithful chromosomal segregation. Therefore, it is not surprising that inherited mutations in key factors involved in the regulation of DNA replication or repair are associated with a range of human diseases hallmarked by the presence of neurodevelopmental abnormalities, neurodegeneration, immunodeficiency, infertility, intellectual disability and cancer predisposition e.g. Ataxia-Telangiectasia (A-T), Nijmegen Breakage Syndrome (NBS), Fanconi anaemia (FA) and Bloom syndrome [9–12].

Post-translational modification (PTM) of DNA replication and repair factors is a key regulatory step that ensures the correct spatiotemporal functioning of the target proteins within the DNA damage response (DDR) pathway. Ubiquitylation is one such extensively studied PTM in which a small protein, ubiquitin (Ub), is attached to target proteins either as a monomer or as chains [13]. During protein ubiquitylation, ubiquitin is covalently linked to a lysine [14] or serine/threonine residues [15] within a target protein by the sequential action of ubiquitin-activating enzymes (E1), ubiquitin conjugating enzymes (E2), and substrate specific ubiquitin ligases (E3) [16]. E3 ubiquitin ligases are able to deposit either a single Ub, termed mono-ubiquitylation, or chains of Ub referred to as poly-ubiquitylation. Deposition of a poly-ubiquitin chain is reliant on the internal lysine residues within ubiquitin (K6, K11, K27, K29, K33, K48 and K63) and the N-terminal methionine residue. These poly-ubiquitin chains can be structured either in a homotypic fashion or in a mixed linkage chain. Functional consequences of ubiquitylation of a target protein can be determined by the type of poly-ubiquitin chains added, which include facilitating protein turnover and regulating protein-protein interactions [17,18]. These ubiquitin marks are plastic and thus a class of enzymes known as deubiquitylases (DUBs) are able to remove mono- and poly-ubiquitylation counteracting any interactions/functions of the modified proteins [19]. As a consequence of the diverse roles of the many proteins that are subject to Ub modification, dysregulation of either deposition or removal of Ub has a wide-ranging impact on cellular and organismal health. These can result in a wide range of pathophysiological conditions including cancer, neurodegeneration, immunodeficiency and neurodevelopmental abnormalities, some of which will be discussed further in this review [20,21].

The highly regulated and crucial process of DNA replication has been increasingly recognised as a significant contributor to many human malignancies and developmental disorders. Here, ubiquitylation plays a central role in regulating DNA replication, which occurs primarily through the modification of histones and components of the replisome, which serves to increase the accessibility of chromatin so that replication can occur, promotes assembly of the replication machinery, mediate intracellular signalling initiated by replication stress and facilitate termination once replication is complete [22]. For example, at the onset of S-phase the Skp2-Cullin-F-box (SCF)-CRL4^CDT2^ E3 ligase ubiquitylates the licencing factor CDT1 to trigger its proteolysis, which ensures no new replication origins are licenced and that the genome is duplicated only once per cell cycle [23]. When a replication fork encounters a bulky distortion or adduct, the E3 ligase RAD18 catalyses the mono-ubiquitylation of PCNA, which promotes the recruitment of trans-lesion synthesis (TLS) polymerases e.g. Polη that can synthesise past a template distorting lesion [24]. Interestingly, PCNA poly-ubiquitylation by HLTF and SHPRH E3 ligases on the same lysine residue modified by RAD18 stimulates template switching to restore DNA replication past a blocking lesion through a mechanism relying on fork remodelling [25–27]. This indicates that mono- or poly-ubiquitylation of the same residue on a protein can have drastically different impacts on its function.

Like its role in replication, protein ubiquitylation plays an important role in regulating DNA repair, which can occur through a variety of different mechanisms, such as facilitating the recruitment or removal of repair factors to/from sites of DNA damage or altering DNA repair protein turnover. For example, the E3 ligase activities of RNF8 and RNF168 are both required for the efficient recruitment of 53BP1 and the BRCA1-BARD1 complex to sites of DNA damage, which subsequently directs repair towards non-homologous DNA end joining (NHEJ) or homologous recombination (HR) respectively [28]. In addition, the BRCA1 E3 ligase is also essential for directing HR-based processes through its ability to promote DNA end resection and loading of the RAD51 recombinase onto the single-stranded DNA (ssDNA) generated by resection [29]. Whilst RNF8, RNF168 and BRCA1 function directly to regulate DSB repair, other E3 ligases like TRIP12, UBR5 and the APC/C impact DNA repair indirectly by controlling the turnover of components of the pathway e.g. RNF168 and CtIP [30,31]. Similarly, in the base excision repair (BER) pathway, the E3 ligase CHIP has been shown to target multiple different BER enzymes for ubiquitylation, which appears to primarily be involved in regulating their stability in unstressed conditions [32]. Interestingly, it has been shown that the induction of damage leads to the formation of stable complexes between XRCC1 and other BER proteins, such as Ligase III and polymerase β, which blocks their association with CHIP thus preventing their ubiquitylation and subsequent degradation [32,33]. In contrast, the RNF4 E3 ligase is thought to be involved in switching off the repair process by enhancing the release of specific DDR proteins, such as MDC1 and PARP1, from chromatin by the p97/VCP segregase [34–36]. Interestingly, RNF4 falls into a unique class of E3 ligases, whose activity is directed by the substrate being previously modified by the ubiquitin-like molecule, SUMO. This growing class of SUMO-targeted E3 ligases or STUbLs, which also contains the Arkadia and Arkadia-like E3 ligases, highlights the interplay between the Ub and SUMO pathways which can have both positive and negative influences on pathway activation and suppression [37]. Together, these examples serve to emphasise the dynamic relationship between DNA damage and the ubiquitin system and the diverse way in which ubiquitin can promote or inhibit different repair pathways.

Since the role of ubiquitylation in regulating the processes of DNA replication and repair have been discussed in depth in reviews elsewhere [22,38–40], this review will focus on ubiquitin E3 ligases known to participate in the maintenance of genomic integrity that are mutated in human genetic disorders (Table 1).

**Table 1.** Selected human genetic disorders associated with inherited mutations in different E3 ubiquitin ligases linked with regulating DNA repair and/or DNA replication.

Protein	Human disorder	Principal clinical phenotype	References
RNF168	RIDDLE	Radiosensitivity, immunodeficiency, dysmorphic features, learning difficulties, short stature and ocular telangiectasia	[42,44,45]
TRAIP	Microcephalic primordial dwarfism (MPD)	Microcephaly, scaphocephaly, long narrow face, micrognathia	[75]
HUWE1	Juberg-Marsidi syndrome, Brooks-Wisniewski-Brown syndrome	Intellectual disability, growth retardation, microcephaly, neurodevelopmental abnormalities, microgenitalism and other physical abnormalities	[101–104]
TRIP12	Clark-Baraitser syndrome and intellectual disability with/without autism	Intellectual disability, macrocephaly, prominent supraorbital ridges, broad nasal tip, prominent lower lip, large ears, obesity, and macroorchidism	[126–129]
FANCL	Fanconi anaemia (FA)	Bone marrow failure, short stature, skin hypo/hyperpigmentation, microcephaly, abnormal kidneys, radial ray defects, hypoplastic/absent thumbs and gastrointestinal/anorectal malformations	[150,152–156]
BRCA1	Fanconi anaemia (FA)	Short stature, microcephaly, intellectual disability, abnormal skin pigmentation, limb defects, congenital heart defects, neurodevelopmental delay, other congenital defects, predisposition for breast and ovarian cancer	[157–160]
RFWD3	Fanconi anaemia(FA)	Radial ray defects, duodenalatresia, absent thumbs, growth restriction, microcephaly, hypoplastic kidney and bone marrow failure	[196]

## RNF168

RNF168 is a RING (Really Interesting New Gene) domain-containing Ub-E3 ligase. Its ubiquitylation activity at the sites of DNA DSBs is required for the recruitment of critical repair factors, such as 53BP1 and the BRCA1-BARD1 heterodimer [41]. RNF168 was first identified as being important for the DDR as part of an siRNA screen aimed at identifying novel factors responsible for the recruitment of 53BP1 to DSBs [42,43]. Apart from its importance as a regulator of DDR protein recruitment to DNA breaks [30], the significance of RNF168-dependent functions on human health became apparent following the identification of biallelic mutations in *RNF168* as the underlying cause of RIDDLE syndrome, which is clinically characterised by the presence of Radiosensitivity, ImmunoDeficiency, Dysmorphic features, and LEarning difficulties [42,44]. Notably, cells from the affected RIDDLE patient displayed significantly reduced levels of RNF168 protein, altered class switch recombination (CSR), decreased 53BP1 and BRCA1 localisation to DNA breaks, and an increased sensitivity to ionising radiation [42,44]. Following the identification of the first RIDDLE syndrome patient, a second affected individual was quickly identified but this time with divergent clinical features, which included short stature, mild ataxia, ocular telangiectasia and immunodeficiency [45]. Interestingly, these clinical features are reminiscent of those exhibited by patients with A-T and A-T-Like Disorder suggesting that RNF168 functions within the same DDR pathway as ATM and the MRN complex (Mre11/Rad50/Nbs1), which subsequently turned out to be the case.

**Figure 1.**
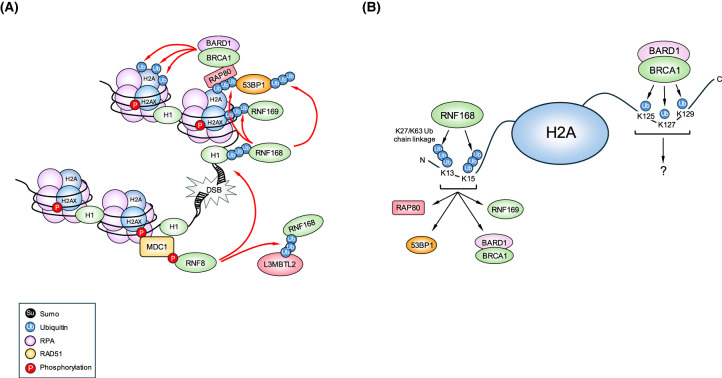
Role of RNF168 is DNA DSB repair. (**A**) RNF168 is recruited to the sites of DSBs by recognition of ubiquitylated Histone H1 and L3MBTL2. Once bound, RNF168 catalyses ubiquitylation of histone H2A and H2A variants, which serve as a recognition signal for 53BP1, BRCA1-BARD1, the BRCA1-A complex and RNF169. (**B**) RNF168 ubiquitylates Lys13/15 located at the N-terminus of H2A(X) to facilitate the recruitment of 53BP1, BRCA1-BARD1, BRCA1-A complex and RNF169. BRCA1-BARD1 mono-ubiquitylates Lys125/127/129 located at the C-terminus of H2A, which function to reposition 53BP1 to promote HR.

RNF168 is composed of three domains: a RING domain and two Ub-dependent DSB recruitment modules, UDM1 and UDM2. The RING domain in RNF168 imparts its E3 ligase function [46], whereas its recruitment to the sites of DSBs is mediated by UDM1 and UDM2. In response to the induction of DSBs, another E3 ubiquitin ligase RNF8, in combination with UBC13 (E2 Ub-conjugating enzyme), catalyses Lys63-linked poly-ubiquitylation of linker histone H1 and L3MBTL2, which serves as the first recruitment signal for RNF168 [47,48] (Figure 1A). In addition to RNF8, HUWE1-mediated ubiquitylation of histone H1 following the induction of UV damage also promotes RNF168 recruitment [49]. However, unlike RNF8 depleted cells, HUWE1 knockout cells do not show a complete abrogation of RNF168 mediated signalling suggesting HUWE1 only plays a supportive role in this pathway. As ubiquitylated substrates, in particular histones, often function as a recruitment platform, the relocalisation of RNF168 to damaged chromatin is mediated by its Ub-binding modules, UDM1 and UDM2. UDM1 (aa 110–188) binds to ubiquitylated H1 downstream of RNF8 [47,48,50], while UDM2 (aa 419–478) helps to reinforce its accumulation by binding to ubiquitylated-H2A and H2A variants, which are the primary substrates of its own E3 ligase activity [47,51]. It has been proposed that leucine-arginine (LR) motif 1 (LRM1) and LRM2 present within UDM1 and UDM2 respectively provide specificity for binding ubiquitylated substrates [47,50].

Once present at the sites of damage, RNF168 catalyses the mono-ubiquitylation of Lys13/15 on H2A/H2AX [52,53], Lys15 on H2AZ and Lys11 on macroH2A1/2 [51] (Figure 1B). Whilst the role of Lys13-Ub on H2A/H2AX is not clear, the ubiquitylation-dependent recruitment motif in 53BP1 and the BRCT-domain-associated ubiquitin-dependent recruitment motif in BARD1 (of BRCA1-BARD1 heterodimer) bind to H2A(X) ubiquitylated on Lys15 to either suppress or promote HR-dependent repair respectively [54,55] (Figure 1A,B). Additionally, the recruitment and retention of 53BP1 at sites of damage has also been shown to require its Lys63-dependent poly-ubiquitylation by RNF168 suggesting that the poly-ubiquitylation of 53BP1 itself can somehow stabilise its binding to mono-ubiquitylated H2A(X) or the surrounding chromatin [56] (Figure 1A). Interestingly, another study demonstrated that RNF168 could also catalyse the formation of Lys27-linked ubiquitin chains on Lys13/15 of H2A/H2AX and that loss of this modification disrupted the ability of cells to recruit 53BP1 and BRCA1 to DNA breaks [57]. However, how the specificity of the E3 ligase activity of RNF168 bound at sites of DNA damage is regulated and whether a substrate is modified by Lys27- or Lys63-linked poly-ubiquitin chains requires further investigation.

Given the critical role that RNF168 plays in recruiting 53BP1 to DNA DSBs and the essential requirement for 53BP1 in mediating the repair of DSBs generated during CSR [58,59], it is likely that the antibody deficiency exhibited by the affected patients is directly caused by an inability to recruit 53BP1 to DNA breaks generated within the immunoglobulin locus during antibody diversification. However, whether this is linked with the neurological deficits also displayed by RIDDLE syndrome patients is not clear.

**Figure 2.**
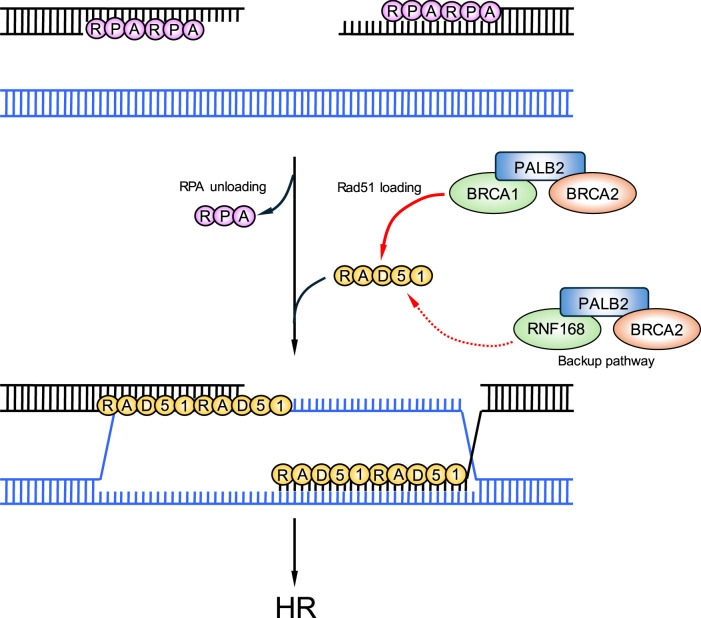
RAD51 loading during HR. BRCA1 forms a complex with PALB2 and BRCA2 to assist in the displacement of RPA and to promote RAD51 loading onto ssDNA. In the absence of BRCA1, the RNF168-PALB2-BRCA2 complex helps to facilitate RAD51 loading onto ssDNA to stimulate HR-dependent repair.

To complicate matters, RNF168 has been reported to catalyse the addition of another ubiquitin-like protein, NEDD8, onto H2A in response to DNA damage. Contrary to its role in promoting H2A ubiquitylation, RNF168-dependent H2A NEDDylation reportedly suppresses its ubiquitylation. Therefore, in response to DSBs, the NEDDylation of H2A decreases over time to facilitate 53BP1 and BRCA1 recruitment [60]. This indicates that RNF168 can both positively and negatively regulate DDR protein recruitment to damaged chromatin by modulating the levels of histone ubiquitylation and NEDDylation. Lastly, it has been shown that RNF168 can form a complex with PALB2 and BRCA2, which can facilitate the loading of RAD51 onto single-stranded DNA in the absence of BRCA1, which is discussed in more detail below [61,62] (Figure 2). This observation demonstrates that RNF168 can promote the use of homology-directed DNA repair by stimulating RAD51 loading either by promoting the recruitment of the BRCA1-A complex to sites of DNA damage or via its interaction with PALB2/BRCA2.

In addition to its role in regulating DNA DSB repair, RNF168 has also been identified as being part of the replisome. It is required for the progression of replication forks in an unperturbed S-phase and the protection of reversed forks from MRE11-mediated degradation [63]. RNF168 mobilisation to the replisome is mediated via its ability to interact with PCNA which requires its E3 ligase activity, ability to bind to the ubiquitylated substrates and a degenerate PCNA-interacting peptide motif present within its C-terminus [63,64]. Whilst the role of RNF168 at replication forks is not clear, it has been shown that overexpressing RNF168 in cells stimulates PCNA ubiquitylation. This suggests that RNF168 may play a role in regulating TLS or template switching under conditions of replication stress [64].

**Figure 3.**
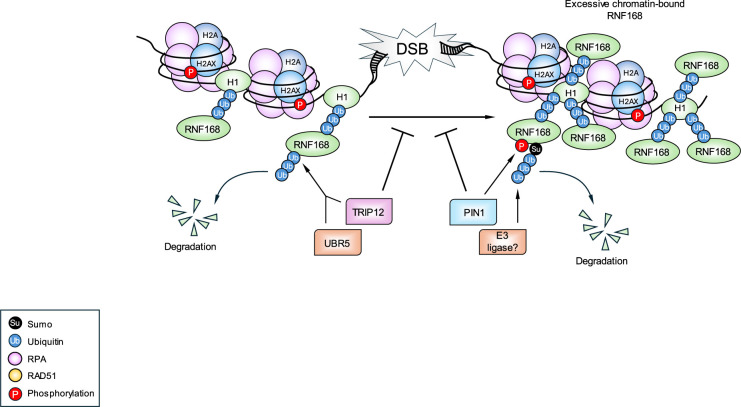
Regulation of RNF168 hyper-accumulation at the sites of damage. TRIP12 and UBR5 poly-ubiquitylate RNF168 to regulate its cellular abundance. In contrast, PIN1 catalysed isomerisation of phospho-RNF168 facilitates its SUMOylation and subsequent ubiquitylation to limit the amount of RNF168 on chromatin surrounding a DSB. Consequently, either an absence of TRIP12/UBR5 or PIN1 results in excessive localisation of RNF168 on damaged chromatin, which compromises DNA DSB repair pathway choice.

Due to its multifaceted role in DNA replication and repair, RNF168 levels are tightly regulated. Indeed, various cancers including breast, ovarian and prostate cancers express high levels of RNF168 that correlate with a poor prognosis and reduced survival rate [65–68]. Cells with high levels of RNF168 are less sensitive to proteotoxic stress coupled with radiation treatment potentially due to their increased ability to recruit 53BP1 to DNA breaks that promotes mutagenic DNA end-joining [67,68]. Conversely, low levels of RNF168 not only give rise to a DSB repair defect but also allows the toxic accumulation of R-loops, which is synthetically lethal with either a BRCA1 or BRCA2 deficiency [65]. Therefore, despite functioning within the same pathway during DSB repair, RNF168 and BRCA1/2 have independent roles in dealing with replication stress, particularly replication stress arising due to unresolved R-loops. In support of this, it was reported that the role of RNF168 in resolving R-loops is mediated in part through its ability to promote the recruitment of the RNA helicase DHX9 to R-loop-prone loci in a ubiquitin-dependent manner [65], whereas the ability of BRCA1 to remove R-loops has been linked with binding the RNA helicase SETX [69]. Thus, to maintain physiological RNF168 levels, its abundance is regulated by two E3 ligases, TRIP12 and UBR5. Consequently, depletion of TRIP12 and UBR5 leads to a large increase in the cellular pool of RNF168 protein and a hyper-accumulation on chromatin surrounding a DSB, which has a deleterious impact on DNA repair pathway choice [30] (Figure 3). It is not clear why merely increasing the total pool of cellular RNF168 would lead to a pathogenic accumulation of it and other downstream factors, such as 53BP1, on chromatin but would suggest that additional pathways exist to modulate the loading and unloading of RNF168 from chromatin. In relation to this, a recent study has indicated that the peptidyl-prolyl cis-trans isomerase NIMA-interacting 1 (Pin1)-dependent SUMOylation of RNF168 activity plays an important regulatory role in removing it from the sites of damage and preventing its hyper-accumulation on chromatin [70] (Figure 3). Interestingly, this study showed that the hyperaccumulation of RNF168 results in increased H2A ubiquitylation and 53BP1 recruitment to DSBs, which is capable of relocating key HR factors, such as BRCA1 and RAD51 to breaks. Independently, another study has suggested that RNF168 SUMOylation facilitates its ability to undergo liquid-liquid phase separation, which can be reversed by SENP1-mediated deSUMOylation [71]. Combined, these studies highlight the complexity of regulating RNF168 function at DSBs and the interplay between ubiquitin- and SUMO-dependent mechanisms in this process. Additionally, more recently described roles for RNF168 in DNA replication underscore its importance both as a DNA repair and DNA replication factor. Therefore, perhaps unsurprisingly, tight regulation of RNF168 is required to prevent the onset of neurodegenerative disease, immunodeficiency or cancer development.

## TRAIP

Microcephalic primordial dwarfism (MPD) is a collective term for a group of related neurodevelopmental disorders, characterised by severe microcephaly and pre- and post-natal growth retardation, which includes Seckel syndrome, Meier-Gorlin syndrome and microcephalic osteodysplastic primordial dwarfism syndrome [72]. MPD is frequently caused by recessive mutations in genes that encode proteins involved in regulating DNA replication, DNA repair, mitotic spindle function and centrosome biogenesis. Although mechanistically distinct, the common clinical phenotypes exhibited by MPD patients are thought to arise from a reduction in cellular proliferation and/or excessive cell death in the developing embryo, which reduces the number of cells available to maintain normal foetal growth [73,74]. Consequently, the affected individuals are often very short and have small heads/brains (microcephaly).

Following whole-exome sequencing of a cohort of patients with MPD, three unrelated individuals were identified with mutations in the gene *TRAIP* [75]. TRAF-interacting protein (TRAIP), also known as RNF206, is another RING domain-containing E3 ubiquitin ligase, which was originally identified as an interactor of TNF receptor-associated factor 1 (TRAF1) and TRAF2 [76]. Members of the TRAF family function as signal transducers of plasma membrane associated receptors and play important roles in regulating the activity of nuclear factor-κB (NFκB), interferon regulatory factors and mitogen-activated protein kinases [77]. Based on this association, it was proposed that once bound to TRAF2, cytoplasmic TRAIP negatively regulates that activation of NFκB in response to TNF-α [76,78]. In contrast with these initial observations, TRAIP was demonstrated to be a nuclear protein associated with the replication machinery via a C-terminal PCNA-interacting peptide (PIP) box [79,80]. Consequently, cells from affected individuals exhibited a marked increase in their doubling time and a delayed progression of S/G2 phase of the cell cycle [75]. More recently it has been shown that TRAIP is specifically required for the termination of active replication forks in mitosis, a process that safeguards cells from undergoing chromosome segregation with under-replicared DNA.

**Figure 4.**
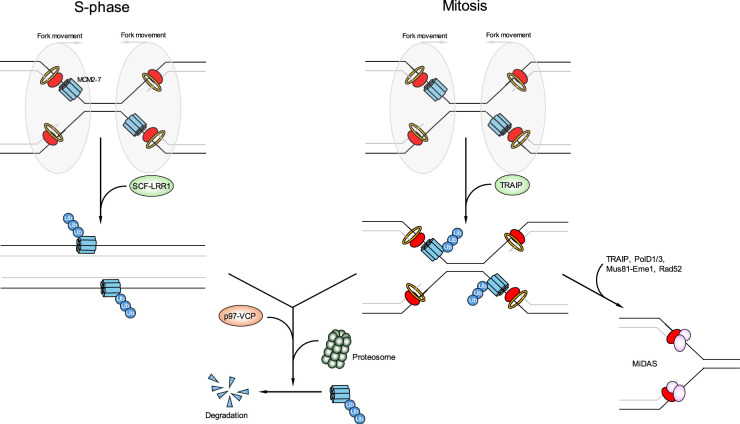
Role of TRAIP during unperturbed replication termination. Unperturbed replication forks converge and bypass one another during S-phase signalling the termination of replication. The MCM7 subunit of terminated CMG's are ubiquitylated by SCF-Cul2^LRR1^, unloaded by the p97 segregase and then undergo proteosome mediated degradation. When under-replicated DNA persists into mitosis, it triggers TRAIP mediated MCM7 ubiquitylation, which signals for p97-dependent MCM unloading and proteasomal degradation. Replication is then completed through the MiDAS pathway, which requires TRAIP, PolD1/3, Rad52, SLX4 and the structure specific endonucleases Mus81-Eme1.

During late S-phase unperturbed replication forks converge and the ubiquitylation of MCM7 by a Skp-Cullin-F-box (SCF)-type E3 ligase containing Cul2 and LRR1 has been shown to be central for triggering p97/VCP-dependent replisome disassembly at completion of DNA synthesis [81,82]. Recently, using cryo-EM the specificity of the Cul2-LRR1 complex for terminating forks has come to light. Prior to origin firing, the binding site of LRR1, which lies at the interface between MCM3 and MCM5, is blocked when in a double MCM hexamer conformation. Furthermore, during replication elongation, spooling of the excluded DNA strand between the zinc finger domains of MCM3 and MCM5 again sterically blocks LRR1 engagement with the MCM hexamer. However, during termination, loss of the excluded DNA strand allows LRR1 engagement with MCM3/5, which triggers MCM7 ubiquitylation and replisome eviction [83,84] (Figure 4). If not completed in the S-phase, DNA synthesis can continue throughout G2 phase and into mitosis, where TRAIP rather than Cul2-LRR1 mediates ubiquitylation of the MCM helicase [85–87]. Why the cell utilises TRAIP instead of Cul2-LRR1 to trigger replisome disassembly outside of S-phase is not known. Unlike Cul2-LRR1, TRAIP is constitutively associated with replisome. However, it has been proposed to require ‘activation’ via Cyclin B1-Cdk1-dependent phosphorylation to catalyse Lys6 and Lys63 poly-ubiquitylation of MCM7 [4,85]. However, whether Cdk1-dependent phosphorylation alters the E3 ligase activity and/or chromatin association of TRAIP or influences substrate binding affinity remains unclear [85]. TRAIP-mediated disassembly of replisome in mitosis is critical for the synthesis of unfinished DNA replication via a pathway known as mitotic DNA synthesis (MiDAS) [88]. Unlike bulk DNA replication that occurs in S-phase, MiDAS does not require a functional replisome and is carried out by a handful of proteins primarily known to regulate DNA repair and the DDR e.g. PolD1/3, RAD51, Mus81-Eme1, Rad52, Polζ and more recently TRAIP (Figure 4) [89].

**Figure 5.**
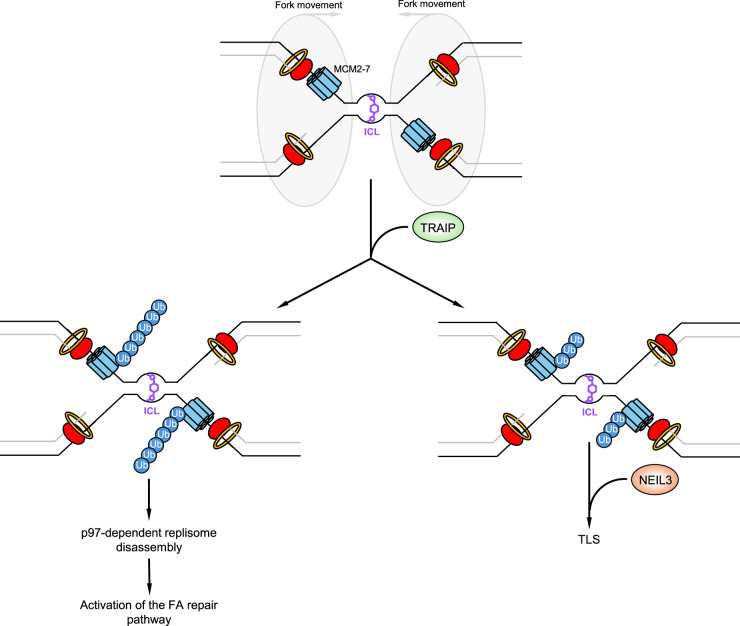
Role of TRAIP during ICL repair. When replication forks converge on an ICL, the stalled CMG's are ubiquitylated by TRAIP. Resolution of the ICL and resumption of DNA synthesis is dictated by the presence of either long or short TRAIP-dependent ubiquitin chains. Longer chains signal the removal of the CMG by p97 and activation of the Fanconi anaemia pathway, whereas shorter ubiquitin chains stimulate recruitment of the DNA glycosylase NEIL3 and resolution of the ICL by the TLS pathway.

In addition to replication termination and MiDAS, it has been suggested that TRAIP and its E3 ligase activity are also involved in dealing with different types of replication stress e.g. R-loops, DPCs and DNA inter-strand cross-links (ICLs). Interestingly, when two replication forks converge at an ICL, TRAIP-dependent ubiquitylation of the CMG complex can facilitate ICL repair either by catalysing short ubiquitin chains to stimulate recruitment of the DNA glycosylase NEIL3 or long ubiquitin chains to promote p97-dependent replisome disassembly and activation of the FA repair pathway (Figure 5) [90]. Similarly, when the replisome encounters a DPC, TRAIP catalyses ubiquitylation of the DPC, which activates the proteasome-dependent, SPRTN-independent, DPC removal pathway [91]. Lastly, TRAIP has also been implicated in the resolution of replication-transcription conflicts. However, the exact mechanism of its action during R-loop removal remains unknown [92]. Moreover, since inherited mutations that compromise R-loop and DPC removal are primarily associated with neurodegeneration [93,94] and accelerated ageing [95,96] respectively, it is not clear how the loss of these functions of TRAIP contributes to the clinical phenotype exhibited by the patients.

Although the function of TRAIP has primarily been studied in response to replication stress, it has been shown that TRAIP also localises to the sites of DNA breaks and is important for DSB repair [97]. This repair function of TRAIP requires both its C-terminus, which mediates binding to another E3 ligase complex containing RNF20-RNF40, and its N-terminus that associates with the BRCA1-A complex component, RAP80. Consequently, depletion of TRAIP or expression of deletion mutants that prevent either its localisation or RAP80 binding compromises HR, BRCA1 loading and radiation resistance [97]. Additionally, TRAIP was also found to be mobilised to the sites of UV-induced DNA damage and its depletion reduced the ATR-dependent phosphorylation of replication protein A (RPA) and H2AX following the induction of UV damage [75,98]. This suggests that TRAIP may also play a role in regulating ATR-dependent intra-cellular signalling induced by replication stress, which would be consistent with the ATR-Seckel-like clinical features exhibited by affected patients.

Given the multi-faceted functions of TRAIP, it would be tempting to speculate that the microcephalic dwarfism exhibited by individuals with biallelic variants in TRAIP is caused by a failure of embryonic cells to complete DNA replication and unload the replication machinery in a timely fashion and/or activate the cellular response to replication stress. This would delay the cell cycle and/or increase cell death arising from the missegregation of chromosomes containing under-replicated DNA, both of which would reduce the number of cells produced during embryonic development. Consequently, this would compromise foetal growth and brain development.

## HUWE1/MULE/Lasu1/ARF-BP1

X-linked intellectual disability (XLID) represents a group of intellectual disability syndromes associated with mutations in genes present on the X-chromosome of which fragile X syndrome (FXS) is the most common [99,100]. Whilst FXS is a trinucleotide repeat expansion disorder linked with the *FMR1* gene, two other XLID syndromes, Juberg-Marsidi syndrome and Brooks-Wisniewski-Brown syndrome are caused by *de novo* or heterozygous inherited mutations in the *HUWE1* gene [101]. Affected males are characterised by the presence of intellectual disability (ID), growth retardation, microcephaly, microgenitalism and other physical abnormalities [101–103]. Surprisingly a few females have also been identified with mutations in *HUWE1* (HECT, UBA and WWE domain-containing protein 1), where X-inactivation was skewed towards the X chromosome containing the WT allele. Similar to affected males, affected females exhibited a range of neurodevelopmental abnormalities, ID, growth retardation and dysmorphic facial features [104].

HUWE1 is a large E3 ubiquitin ligase. Structurally, the N-terminal region of HUWE1 contains a ubiquitin-associated domain (UBA) and a WWE domain, both of which are important for Ub-dependent proteolysis. Interestingly, WWE domains have been reported in other E3 ligases to bind poly-ADP-ribose (PAR) chains [105]. However, whether or not HUWE1 is a PAR-directed E3 ligase is currently unknown. A BH3 domain, located in the centre of the protein is thought to regulate substrate specificity, while its C-terminal region harbours a HECT domain, which is responsible for its ubiquitin E3 ligase activity [106]. HUWE1 can catalyse both mono- and poly-ubiquitylation on its substrates (either Lys6, Lys48 or Lys63 chains) [106,107]. Interestingly, HUWE1 has also been shown to catalyse Lys48/Lys63 or Lys11/Lys48 heterotypic ubiquitin chains in cooperation with other E3 ligases. For example, in response to IL1β, HUWE1 co-operates with TRAF6 to catalyse Lys48 linked chains on substrates primed with Lys63 ubiquitylation resulting in the amplification of NF-κB signalling [108]. Similarly, HUWE1 catalyses Lys48/Lys63 branched chains in cooperation with ITCH to regulate the cellular abundance of the tumour suppressor TXNIP [109].

Over the years, numerous substrates of HUWE1 have been identified, implicating it in regulating a diverse range of cellular processes including, cellular proliferation and differentiation, apoptosis, inflammation and the response to DNA damage [106,110]. HUWE1-dependent proteolysis has been shown to be particularly important for maintaining genome stability, for example, HUWE1 has been identified to interact with BRCA1 and promote its degradation [111]. Consequently, loss of HUWE1 results in an increase in BRCA1 protein abundance, which enhances HR-dependent DNA DSB repair and promotes resistance to ionising radiation [111]. Similarly, a decrease in HUWE1 expression suppresses the hypersensitivity of cells harbouring the BRCA1-Δ11q splice variant to PARP inhibitors by stabilising the truncated BRCA1 protein arising from *BRCA1* mis-splicing [112].

In addition to regulating the stability of BRCA1, HUWE1 has been shown to ubiquitylate H2AX and regulate its turnover via proteasomal degradation. Interestingly, it was reported that the ATM-dependent phosphorylation of H2AX in response to the induction of DNA DSBs blocks HUWE1-dependent ubiquitylation, to facilitate DNA repair [113]. This highlights the complex interplay between different PTMs, such as phosphorylation and ubiquitylation at the sites of DNA damage. To further complicate matters, HUWE1 also catalyses the ubiquitylation of histone H1, which is thought to prime the histone for further poly-ubiquitylation by RNF8. This ubiquitylation reaction then facilitates the recruitment of RNF168 and 53BP1 to sites of UV damage [49]. This indicates that HUWE1 has mechanistically distinct roles in regulating HR in response to different type of DNA damage i.e. HUWE1 negatively regulates HR-dependent DSB repair by promoting the degradation of BRCA1, whereas in response to UV damage it promotes the recruitment of RNF168 and 53BP1 to suppress HR. Akin to RNF168, HUWE1 has also been suggested to act as a NEDD8 E3 ligase. Interestingly, in contrast with its role in regulating HR, HUWE1-dependent NEDDylation facilitates NHEJ-mediated DSB repair by stimulating the autophosphorylation and activation of DNA-dependent protein kinase catalytic subunit (DNA-PKcs) [114].

In addition to DNA repair, HUWE1 has also been implicated in the cellular response to replication stress. Depletion of HUWE1 leads to cell cycle arrest in S-phase and increases cellular sensitivity to replication stress inducing agents such as hydroxyurea [115]. In keeping with a direct role for HUWE1 in regulating the replication stress response, it is recruited to stressed replication forks, via an interaction with PCNA using a C-terminal PIP box motif, and mono-ubiquitylates H2AX [115]. Intriguingly, under conditions of replication stress, the HUWE1-dependent ubiquitylation of H2AX appears to be important for BRCA1 and BRCA2 recruitment to damaged chromatin to maintain fork stability [115]. This observation is in stark contrast with the previous reports linking HUWE1 with regulating H2AX and BRCA1 protein stability in unstressed cells. Therefore, whilst HUWE1 is obviously important for coordinating the DDR, the type of genotoxic insult dictates the mechanism by which it performs its functions.

Additionally, HUWE1 has been shown to be involved implicated in regulating the ATR replication stress response through the degradation of the key factors, 53BP1 and CHK1 [116,117]. This suggests that HUWE1 may play a critical role in down-regulating the ATR-dependent checkpoint response to facilitate restoration of DNA replication once the damage has been repaired. Notably, HUWE1 has also been reported to mediate the degradation of CDC6, a component of pre-replication complex responsible for loading the MCM helicase onto replication origins, in response to DNA damage [118]. This has been proposed to represent a fail-safe mechanism to ensure that origin relicensing does not occur and highlights another example of how HUWE1 plays a critical role in down-regulating multiple DDR pathways.

Lastly, HUWE1 has been shown to be involved in modulating the stability of components of the BER pathway. Damaged bases can be corrected by the BER pathway, which utilises the sequential action of different enzymes to excise the damaged base and fill in the gap. The gap filling step can be carried out by one of two DNA polymerases, DNA polymerase beta (Pol β) and DNA polymerase lambda (Pol λ), both of which have been identified as substrates of HUWE1 [119,120]. In both cases, HUWE1 regulates the steady state turnover of both Pol β and Pol λ. However, in the case of Pol λ, the HUWE1-dependent degradation is suppressed by Pol λ phosphorylation following the induction of oxidative damage, which facilitates its association with chromatin [120]. Interestingly, it was reported that cells from XLID patients with mutations in HUWE1 exhibited an increased mutation rate and hypersensitivity to oxidative damage. Strikingly, in the case of the p.R4187C patient-associated HUWE1 mutation, this increased its E3 ubiquitin ligase activity, which consequently lead to a decrease in Pol λ levels and BER capacity [121].

Inherited mutations in HUWE1 contribute to a diverse constellation of clinical features. This is underscored by the distinct impact these mutations have on the regulation of various DNA repair and replication stress response pathways.

## TRIP12

Thyroid hormone Receptor Interacting Protein 12 (TRIP12) is another HECT domain containing Ub-E3 ligase. Similar to HUWE1, TRIP12 has been implicated in regulating a multitude of cellular processes including cell cycle progression, differentiation, intracellular signalling, gene expression and the DDR [122,123]. Its importance during development is supported by the embryonic lethality of mice bearing E3 ligase-inactivating mutations [124]. Embryonic stem cells obtained from these mice show decreased cell proliferation and altered chromatin remodelling while maintaining an ability to differentiate [124]. Interestingly, TRIP12 has been shown to be important for proteolysis-targeting chimeras mediated degradation of specific substrates by catalysing Lys29 linked chains leading to the formation Lys29/Lys48 branched Ub chains [125].

Mutations in *TRIP12* have been linked with several syndromic and non-syndromic neurodevelopmental deficits including Clark-Baraitser syndrome and intellectual disability with/without autism [126–129]. Patients with Clark-Baraitser syndrome display intellectual disability, macrocephaly, prominent supraorbital ridges, broad nasal tip, prominent lower lip, large ears, obesity, and macroorchidism.

Interestingly, TRIP12 was identified along with another Ub-E3 ligase UBR5 as part of an siRNA screen aimed at discovering novel factors involved in regulating the accumulation of RNF168 at sites of DNA damage [30]. Strikingly, depletion of TRIP12 and UBR5 lead to the supraphysiological accumulation of RNF168 and 53BP1 on chromatin surrounding a DSB, which was partly attributed to TRIP12 and UBR5 playing a role in regulating the steady state levels of RNF168 (Figure 3) [30]. However, whether TRIP12 and UBR5 are also involved in the direct removal of RNF168 from DSBs is not clear. Similar to RNF168, TRIP12 has been shown to target PARP1 for proteasomal degradation, which is thought to limit the levels of PARylation induced by DNA damage [130]. The interaction between TRIP12 and PARP1 is mediated by its WWE domain, which allows TRIP12 to interact with and degrade PARP1 only when it is PARylated [130]. This function renders cells lacking TRIP12 highly sensitive to PARP inhibitors as it results in elevated levels of PARP1 trapped on DNA [130]. Notably, TRIP12 has been shown to stably interact with the DUB USP7 via its AWL domain. This serves to protect TRIP12 from auto-ubiquitylation-dependent degradation [131]. Consequently, cells lacking USP7 are hypersensitive to a variety of DNA damaging agents [132]. However, since USP7 has been implicated in controlling the stability of many different E3 ubiquitin ligases associated with DNA repair and replication, including Rad18, HLTF, RNF168, RNF169 and UHRF1, it is not clear how much decreased TRIP12 stability contributes to the DNA damage phenotype associated with loss of USP7 [133–138]. Lastly, it has been suggested that TRIP12 also plays a role in controlling spindle assembly checkpoint (SAC) activation [133]. However, whilst the underlying mechanism relating to how TRIP12 regulates SAC activation is unclear, depletion of TRIP12 prolongs the metaphase-to-anaphase transition, which gives rise to chromosome segregation defects via mitotic slippage.

Although TRIP12 is clearly important for the proper regulation of several different DNA damage and replication stress pathways, it is not evident how loss of this control in cells from patients with mutations in *TRIP12* contributes to the clinical presentation of the disease. Variants in genes associated with DNA damage repair or DNA replication pathways often present with growth retardation, a reduction in head/brain size and skeletal defects, none of which are typically exhibited by individuals with Clark-Baraitser syndrome. Whilst it is possible that many of the developmental defects linked with DNA repair or replication abnormalities are suppressed by the presence of a remaining WT *TRIP12* allele in Clark-Baraitser syndrome patients, only a systematic analysis of the DDR- and replication-related functions of TRIP12 in patient cells will help define which contribute to the disease phenotype.

## FANCL

FA is predominantly a bone marrow failure syndrome associated with other congenital abnormalities, such as microcephaly, short stature, cardiac and skeletal anomalies, radial ray malformations (abnormal thumb and radius), skin abnormalities, osteoporosis, abnormal kidney structure, genitourinary and gastrointestinal malformations [139,140]. FA also predisposes patients to various cancers including but not limited to acute myeloid leukaemia, head and neck squamous cell carcinoma, skin, breast and ovarian cancer and Wilm's tumour [139]. FA occurs worldwide and affects ∼1:100 000–300 000 people. FA is caused by mutations in one of at least 22 different genes (FANCA-FANCW) all of which are involved in DNA damage repair and maintaining genome stability (reviewed earlier [8,140]). Historically, FA genes have been specifically associated with repairing DNA intra-/inter-strand cross-links (ICLs) induced by genotoxins such as, cisplatin, mitomycin C and diepoxybutane, that interfere with DNA replication [141]. However, more recently, it has been shown that FA genes may have evolved to deal with ICLs and DPCs induced by naturally occurring aldehydes e.g. formaldehyde or acetaldehyde, generated as byproducts from alcohol metabolism, lipid peroxidation and DNA demethylation [8].

Activation of the FA pathway is a multi-step process primarily initiated by two replication forks converging upon an ICL. Fork convergence triggers ubiquitylation of the two CMG complexes by the TRAIP E3 ubiquitin ligase and their removal by the p97 ATPase (Figure 5). This allows one of the forks to undergo reversal, which may be carried out by the FANCM-MHF1/2 complex or another fork reversal enzyme. Collision of a fork with an ICL triggers activation of the FA core complex, which contains FANCA, FANCB, FANCC, FANCE, FANCF, FANCG, FAAP20, FAAP100, the E3 ubiquitin ligase FANCL and the E2 ubiquitin conjugating enzyme UBE2T/FANCT [142,143]. Activation of the FA core complex stimulates monoubiquitylation of a protein clamp-like complex consisting of FANCD2-FANCI (ID complex), which is facilitated by the phosphorylation of FANCI by ATR [144]. This allows the ID complex to stably encircle the DNA containing the ICL [145,146]. It has been proposed that the DNA-bound mono-ubiquitylated ID complex functions as a platform for the recruitment of enzymes involved in excision of the ICL e.g. SLX4/FANCP, Mus81, XPF/FANCQ and SLX1, and subsequent repair of the two damaged DNA strands by components of the HR repair pathway.

FANCL is a RING domain containing Ub-E3 ligase with an N-terminal E2-like fold (ELF) domain and a central double RWD (DRWD) domain [147]. The ELF domain of FANCL interacts with ubiquitin to regulate FANCD2 mono-ubiquitylation *in vivo*, while the DRWD and RING domains directly interact with the FANCD2/FANCI complex and UBE2T/FANCT respectively [148–150]. FA complementation group L is a rare subgroup and has only been diagnosed in a few patients [151–157]. Typically, affected patients display clinical symptoms commonly used as diagnostic markers of FA, including bone marrow failure, short stature, skin hypo/hyperpigmentation, microcephaly, abnormal kidneys, radial ray defects, hypoplastic/absent thumbs and gastrointestinal/anorectal malformations [151–157]. Consistent with the known role for FANCL in mediating FANCD2/I ubiquitylation, cells from the affected patients exhibit a complete loss of damage inducible FANCD2/I ubiquitylation and a hypersensitivity to DNA cross-linking agents. Based on this, all the clinical phenotypes exhibited by the patients can be explained by an absence of DNA- and protein-cross-link repair.

## BRCA1

Despite it being well established that heterozygous mutations in *BRCA1* predispose women to develop breast and ovarian cancer at a very early age, only recently was it discovered that biallelic mutations in *BRCA1* cause a FA-like neurodevelopmental disorder designated complementation group S (FANCS). Affected individuals exhibit many of the clinical features typically associated with FA such as short stature, microcephaly, intellectual disability, abnormal skin pigmentation, limb defects, congenital heart defects, neurodevelopmental delay and other congenital defects [158–161]. However, bone marrow failure is generally not observed. Interestingly, whilst affected individuals have an increased predisposition for developing breast and ovarian cancer, other cancer types, such as T-cell acute lymphoblastic leukaemia (T-ALL) and neuroblastoma have been observed [158,160,161]. Cells from FANCS patients have decreased levels of BRCA1 protein, fail to form DNA damage-induced BRCA1 and RAD51 foci and exhibit a hypersensitivity to DNA cross-linking agents [158,160].

BRCA1 is a RING domain containing Ub-E3 ligase that is central to the HR DNA repair pathway [29]. The N-terminal region of BRCA1 contains the RING domain, which interacts with BRCA1-associated RING domain protein 1 (BARD1) to form a heterodimeric E3-ligase [162,163]. In S/G2 phase of cell cycle, BRCA1-BARD1-mediated H2A ubiquitylation is required for 53BP1 repositioning to facilitate end-resection, a pivotal process that directs DNA DSB repair towards HR-dependent pathways [164]. This is in part due to the ability of BRCA1-BARD1 to facilitate the mono-ubiquitylation of three lysine residues (Lys125/127/129) located within the C-terminus of histone H2A [165,166] (Figure 1B). However, the requirement of the BRCA1-BARD1 E3 ligase activity for HR and its role as a tumor suppressor remains controversial [167–171]. Knock-in mice bearing the Ile26Ala BRCA1 RING domain mutation (which compromises its E3 ligase activity but retains the ability to form heterodimer with BARD1) did not display any overt developmental defects or increased predisposition for developing tumours [168]. Consistent with this, embryonic stem cells or mouse embryonic fibroblasts from the BRCA1 Ile26Ala knock-in mutant mice did not exhibit any increases in chromosomal stability or defects in cell proliferation, HR efficiency or RAD51 loading at the sites of damage [167,168]. However, it is important to note that the auto-ubiquitylation activity of BRCA1 Ile26Ala mutant was used as a readout of its ligase function rather than its ability to ubiquitylate its substrate, histone H2A [168]. In contrast, knock-in mice bearing the patient-associated, BRCA1 RING domain mutation, Cys61Gly, which compromises both its ability to interact with BARD1 and E2 conjugating enzyme, are embryonic lethal similar to a complete loss of BRCA1 [169]. However, heterozygous BRCA1 Cys61Gly mutant mice with tissue-specific loss of p53 develop mammary carcinomas resembling BRCA1 null tumours, although these tumours rapidly developed resistance to cisplatin and PARP inhibitors [170]. Consistent with this, whilst cells expressing the Cys61Gly BRCA1 mutant displayed a significant reduction in their ability to carry out HR as determined using a reporter assay, they were still able to form DNA damage-induced RAD51 foci [169], indicating that this mutation is hypomorphic. More recently, a mutation in the RING domain of BARD1 was identified, Arg99Glu, that was shown to compromise the E3 ligase activity of BRCA1-BARD1 complex without impacting the stability of the complex [164]. Interestingly, cells complemented with the Arg99Glu BARD1 mutant displayed a hypersensitivity towards certain DNA damaging agents, such as Olaparib and cisplatin. Consistent with a role for the E3 ligase activity of the BRCA1/BARD1 complex being important for repair, cells expressing the Arg99Glu BARD1 mutant failed to efficiently form DNA damage-induced RAD51 foci, which was associated with a concomitant reduction in HR. Notably, it was shown that this HR defect could be restored by transfecting the cells with an H2A-ubiquitin fusion, strongly indicating that the ability of BRCA1/BARD1 to ubiquitylate histone H2A is required for its ability to drive HR-dependent DSB repair [164]. Despite this, a recent report suggests that both the Ile26Ala BRCA1 and R99E BARD1 mutants do not completely lack ligase activity towards H2A, thus complicating interpretations of the earlier reports [172]. Importantly, it was revealed that assessment of the E3 ubiquitin ligase activity of the BRCA1/BARD1 complex critically depended on which E2 conjugating enzymes were used in the *in vitro* ubiquitylation reactions and whether H2A was presented in the context of a nucleosome or not [172]. In the same study, the authors identified a combination of mutations in BRCA1 (Ile26Ala, Leu63A, Lys65Ala) that completely abrogated the E3 ligase activity of the BRCA1-BARD1 complex towards nucleosomes irrespective of the E2 conjugating enzyme used [172]. Critically, using the BRCA1^I26A/L63A/K65A^ mutant, it was demonstrated that the E3 ligase activity of the BRCA1/BARD1 complex is essential for DNA DSB end-resection, RAD51 localisation, HR-dependent repair and cellular resistance towards cisplatin and Olaparib [172]. However, the impact of this mutant on tumour suppression remains to be determined.

In addition to the mono-ubiquitylation of H2A, several reports suggested that BRCA1 can also catalyse atypical Lys6-linked poly-ubiquitin chains at sites of DNA damage. However, what substrate(s) is targeted for BRCA1-dependent Lys6 ubiquitylation remains unclear [173–175].

In addition to its role in early steps of DSB repair by HR, BRCA1 forms a complex with PALB2 and BRCA2 (BRCA1-PALB2-BRCA2 complex) to assist in loading the RAD51 recombinase onto single-stranded DNA [176,177] (Figure 2). BRCA1 is present in several multiprotein complexes, termed the BRCA1-A, B and C complexes, which are largely defined by the proteins that differentially bind to two C-terminal BRCT domains of BRCA1 [178]. The BRCA1-A complex contains the Ub-binding protein RAP80, Abraxas, MERIT40, BRCC45 and the DUB BRCC36 [179]. The presence of a tandem ubiquitin interacting motif and SUMO interacting motif in RAP80 mediates localisation of the BRCA1-A complex to sites of damage [178,180,181] (Figure 1). Interestingly, the presence of the DUB BRCC36 in this complex has been suggested to fine-tune the level of chromatin ubiquitylation at sites of DNA breaks to prevent excessive end-resection [182]. The BRCA1-B complex consists of TOPBP1 and BACH1/FANCJ and has been shown to play a role in DNA replication, ICL repair, and S-phase progression [183,184]. Lastly, the BRCA1-C complex consists of CtIP and the MRN complex, which directly carries out DNA DSB end-resection and RPA loading to facilitate HR [178,185].

The importance of BRCA1 in maintaining genome stability can also be highlighted by the fact that in addition to DSB repair, BRCA1 also protects damaged replication forks from uncontrolled nucleolytic attack [186]. This way BRCA1 supports cell survival under conditions of replication stress caused by nucleotide imbalances or when a replication fork encounters a DNA lesion, secondary DNA structure, mis-incorporated ribonucleotides or the transcriptional machinery [187–189]. Recent work suggests that the ability of BRCA1 to protect stressed replication forks also relies on its ability to load RAD51 onto single-stranded DNA (ssDNA) at reversed replication forks [187,190]. Lastly, BRCA1 is also known to be critical for resolving RNA:DNA hybrids or R-loops by recruiting the RNA helicase Senataxin (SETX) [69].

Given the diverse roles that BRCA1 plays in maintaining genome stability in response to the induction of DNA DSBs and replication stress, it is unsurprising that FANCS patients exhibit clinical deficits typically observed in individuals with inherited mutations in other genes with known roles in repairing damaged DNA. Moreover, since biallelic mutations in other critical HR factors, such as BRCA2/FANCD1, PALB2/FANCN, RAD51/FANCR, RAD51C/FANCO and XRCC2/FANCU have been identified as an underlying cause of an FA-like phenotype in other patients, this highlights the importance of the HR pathway in dealing with impediments to DNA replication during embryonal development caused by naturally occurring DNA inter-/intra-strand and DNA/protein-cross-links [191–196].

## RFWD3

Compound heterozygous mutations in *RFWD3* have been identified in a single child with a FA-like phenotype. The proband presented with clinical features typically associated with FA, such as radial ray defects, duodenal atresia, absent thumbs, growth restriction, microcephaly, hypoplastic kidneys, bone marrow failure and a cellular hypersensitivity to DNA cross-linking agents [197]. RFWD3 is the most recent FA gene to be identified and has been designated complementation group W (FANCW). Consistent of this designation, *Rfwd3* knockout mice exhibit similar features to other FA gene knockouts, such as a shorter life span, infertility and a hypersensitivity to mitomycin C [198].

RFWD3 is the third RING domain containing Ub-E3 ligase identified to function within the FA pathway and has been shown to bind and ubiquitylate RPA [199–202]. RPA is a trimeric complex, compromised of a 70, 32 and 14 kDa subunit, that binds to ssDNA generated during unperturbed replication, upon replication fork stalling or DNA DSB end-resection. This not only functions to protect ssDNA from nucleases but facilitates the recruitment/activation of other factors involved in DNA repair and replication [203]. RFWD3 interacts with the WD40 domain of RPA2 facilitating its recruitment to the sites of damage [200]. Interestingly, the RFWD3 missense mutation (p.Ile639Lys) identified in the affected proband localised within the WD40 domain and compromised the ability of RFWD3 to interact with RPA, highlighting the functional importance of this interaction [200]. It has been shown that RFWD3 ubiquitylates RPA bound to stalled replication forks, which increases its turnover to promote fork restart and HR [200–202]. Additionally, RFWD3 also ubiquitylates RAD51, which facilitates its extraction from DNA by p97/VCP to aid in steps of HR downstream of RAD51 loading [201]. Unsurprisingly, depletion of RFWD3 or complementation of FANCW patient-derived cells with an RPA-interaction deficient mutant sensitises cells to MMC, camptothecin or Olaparib [197,200]. Surprisingly, it has been reported that loss of RFWD3 in cells depleted of BRCA2 rescues the hypersensitivity to genotoxins that stall replication and subsequent fork degradation and collapse [204]. It has been proposed that the loss of RPA ubiquitylation caused by an absence of RFWD3 allows cells to load small amounts of RAD51 onto reversed forks in a BRCA2-independent manner, protecting them from Mre11-dependent degradation. In contrast with this model, it has recently been reported that RFWD3 catalyses the poly-ubiquitylation of PCNA promoting recruitment of the replication fork remodelling factor ZRANB3 to stressed forks [205]. From these observations, it was suggested that the loss of ZRANB3-dependent fork reversal in BRCA2 depleted cells lacking RFWD3 underlies the mechanism with which loss of RFWD3 can restore the stability of deprotected forks. In addition to its role in the FA pathway, RFWD3 has also been shown to promote DNA damage tolerance by TLS through its ability to potentiate the ubiquitylation of Lys164 on PCNA and facilitate recruitment of TLS polymerases to damaged replication forks [206,207]. Interestingly, the RFWD3 missense mutation (p.Ile639Lys) identified in the affected proband also affected PCNA ubiquitylation, therefore it is difficult to dissect whether it is the loss of RPA interaction or the reduction in PCNA ubiquitylation that contributes more to the development of a Fanconi phenotype [189]. In a manner similar to RNF168, RFWD3 has a degenerate PIP motif located within its C-terminus, which mediates its recruitment to the replication machinery and is essential for maintaining DNA synthesis [208]. Interestingly, this degenerate PIP motif is located within its WD40 domain near the FANCW patient associated missense variant. Consequently, the p.Ile639Lys mutation reduces the ability of RFWD3 to bind to PCNA [208].

Based on the critical role that RFWD3 plays in regulating the localisation of RPA, RAD51 and components of the TLS pathway to damaged replication forks, it is unsurprising that mutations in RFWD3 give rise to an FA-like phenotype. Although, it has not been formally shown that RFWD3 is also important for dealing with more physiological DNA cross-links and DPCs induced by naturally occurring genotoxic metabolites, such as aldehydes, it seems likely that the clinical deficits exhibited by the affected patient are caused by an inability to deal with these lesions during embryogenesis.

## Perspectives

Whilst it is clear that the ubiquitin/SUMO pathway is critical for regulating both DNA replication and DNA repair, the contribution that disrupting this regulatory pathway has on the development of disease in humans is only just becoming apparent. Due to the increasing affordability of whole exome and whole genome sequencing, patients with rare, often orphan genetic disorders, are being more frequently identified. As a consequence, it is highly likely that other E3 ubiquitin ligases implicated in regulating DNA replication and repair, such as Rad18, HLTF, RNF4, RNF8, RNF20/40, RNF111/Arkadia, RNF169, components of SCF-type family of E3 ligases and their corresponding de-ubiquitylating enzymes will be identified as disease-causing genes. Since many of these ubiquitin regulating enzymes are essential genes, the identification of hypomorphic mutations associated with specific disease phenotypes will provide invaluable information as to how loss of specific functions affects the development and maintenance of different organs and tissue types. This will undoubtedly implicate the ubiquitin system in regulating unexpected aspects of DNA replication and the cellular DDR that may open up more opportunities for therapeutic intervention.

## Open Access

Open access for this article was enabled by the participation of University of Birmingham in an all-inclusive *Read & Publish* agreement with Portland Press and the Biochemical Society under a transformative agreement with JISC.

## Abbreviations

BER: base excision repair
CSR: class switch recombination
DDR: DNA damage response
DPC: DNA-protein cross-link
DSB: double strand break
DUB: deubiquitylase
ELF: E2-like fold
FA: Fanconi anaemia
FXS: fragile X syndrome
HR: homologous recombination
MiDAS: mitotic DNA synthesis
MPD: microcephalic primordial dwarfism
NHEJ: non-homologous DNA end joining
PCNA: proliferating cell nuclear antigen
PIP: PCNA-interacting peptide
PTM: post-translational modification
SAC: spindle assembly checkpoint
SSB: single strand break
TLS: trans-lesion synthesis
Ub: Ubiquitin
XLID: X-linked intellectual disability
